# The 1-Year Safety and Efficacy Outcomes of Magmaris, Novel Magnesium Bioresorbable Vascular Scaffolds in Diabetes Mellitus Patients with Acute Coronary Syndrome

**DOI:** 10.3390/jcm10143166

**Published:** 2021-07-18

**Authors:** Adrian Włodarczak, Magdalena Łanocha, Marek Szudrowicz, Mateusz Barycki, Alicja Gosiewska, Jan Jakub Kulczycki, Maciej Lesiak, Adrian Doroszko, Piotr Rola

**Affiliations:** 1 Department of Cardiology, The Copper Health Centre (MCZ), 59-300 Lubin, Poland; wlodarczak.adrian@gmail.com (A.W.); marek.szudrowicz@gmail.com (M.S.); jan.jakub.kulczycki@gmail.com (J.J.K.); 2 Adalbert’s Hospital, 61-144 Poznan, Poland; magdalena.lanocha@ump.edu.pl; 3 Department of Cardiology, Provincial Specialized Hospital in Legnica, Iwaszkiewicza Str. 5, 59-220 Legnica, Poland; mateusz.barycki@gmail.com; 4 Faculty of Mathematics and Information Science, Warsaw University of Technology, 00-662 Warsaw, Poland; alicjagosiewska@gmail.com; 5 1st Department of Cardiology, Poznan University of Medical Sciences, 61-491 Poznan, Poland; maciej.lesiak@skpp.edu.pl; 6 Department of Internal Medicine, Hypertension and Clinical Oncology, Wroclaw Medical University, 50-556 Wroclaw, Poland; adrian.doroszko@gmail.com

**Keywords:** diabetes mellitus (DM), acute coronary syndrome (ACS), magnesium bioresorbable scaffold (BRS), Magmaris, percutaneous coronary intervention (PCI), coronary artery disease (CAD)

## Abstract

Background: Diabetes mellitus (DM) is one of the major risk factors contributing to Acute Coronary Syndromes (ACS) and is associated with an increased risk of adverse clinical outcomes following percutaneous coronary intervention (PCI), even when the second generation of drug-eluting stents (DES) is used. In order to overcome the disadvantages of permanent caging of a vessel with metallic DES, bioresorbable scaffold (BRS) technology has been recently developed. However, the prognosis of patients with DM and ACS treated with PCI via subsequent implantation of Magmaris (Biotronik, Berlin, Germany)—a novel magnesium-bioresorbable scaffold—is poorly investigated. Methods: A total of 193 consecutive subjects with non-ST elevation acute coronary syndrome (NSTE-ACS) who, from October 2016 to March 2020, received one or more Magmaris scaffolds were enrolled in this study. The diabetic group was compared with non-diabetic subjects. Results: There were no significant differences in the occurrence of primary endpoints (cardiovascular death, myocardial infarction, and in-stent thrombosis) and principal secondary endpoints (target-lesion failure, scaffold restenosis, death from any reason, and other cardiovascular events) between the two compared groups in a 1-year follow-up period. Conclusions: The early 1-year-outcome of magnesium bioresorbable scaffold (Magmaris) seems to be favorable and suggests that this novel BRS is safe and effective in subjects with NSTE-ACS and co-existing DM.

## 1. Introduction

Acute coronary syndrome (ACS), despite advances in pharmacological treatment and revascularization techniques, remains a significant cause of mortality and morbidity worldwide. Diabetes mellitus (DM) is one of the major risk factors of coronary artery disease (CAD), and its prevalence is still increasing among developed and developing countries [1]. Although the new generation of drug-eluting stents improved the outcomes of percutaneous coronary intervention (PCI), there is still a higher rate of major cardiovascular adverse events in diabetic patients [2], which is related to a disturbance in the artery healing caused by the presence of a metallic scaffold in the lumen of the vessel. It results in the activation of a chronic local inflammation and abnormalities in the vessel architecture with coexisting impairments in vasomotor function [3,4]. To counteract these processes, the first bioresorbable scaffolds (BRS) were introduced more than 10 years ago. The main concept of this vessel-supporting technology was to provide short-term equivalent performance to existing drug-eluting stents (DES), avoiding permanent caging of the vessel due to reabsorption of the scaffold after complete vessel healing and leading to the restoration of normal vascular. Shortly after its appearance on the market, the Absorb scaffold (Abbott Vascular, Santa Clara, CA, USA) became the leading BRS. However, optimism associated with the preliminary studies was restrained by results of the ABSORB II trial [5], and due to safety concerns, this technology was withdrawn from commercial use and is restricted nowadays only to clinical trials. Despite initial difficulties, the BRS concept continues to evolve, and a novel promising magnesium bioresorbable scaffold—Magmaris (Biotronik, Berlin, Germany)—has been recently introduced on the market [6]. Recently published data suggest that the use of Magmaris BRS in patients with ACS is associated with favorable early clinical outcomes [7]. Since particularly diabetes is a predictor of device-related thrombosis and target-lesion failure in the Absorb scaffold [8], we designed this trial in order to evaluate the early (1-year) outcome of patients with diabetes treated with Magmaris.

## 2. Materials and Methods

### 2.1. Study Population

**Figure 1:**
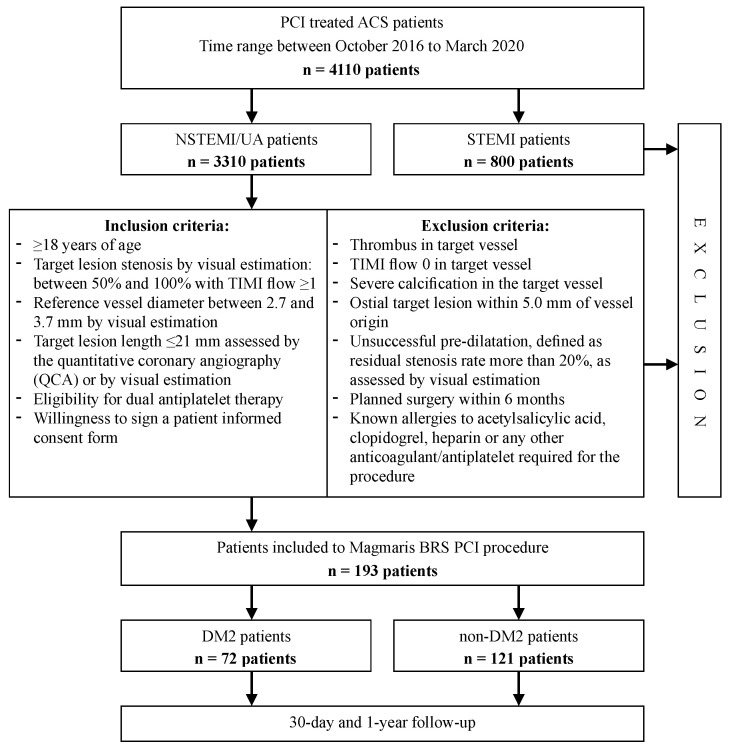
Study procedure, and inclusion and exclusion criteria. Abbreviations: NSTEMI—non-ST elevation myocardial infraction; UA—unstable angina; STEMI—ST elevation myocardial infraction; PCI—percutaneous coronary intervention; TIMI—thrombolysis in myocardial infraction; BRS—bioresorbable scaffolds; DM2—diabetes mellitus type 2.

This investigator-initiated, single-center, single-arm observational study enrolled consecutive 193 patients with no ST-Elevation Myocardial Infarction (NSTEMI) or unstable angina (UA) who received one or more Magmaris BRS at the Cardiology Department of the Cooper Health Center in Lubin between October 2016 and March 2020 during the initial PCI. The study population was selected out of all consecutive patients with ACS who qualified for PCI at the department. A total of 4110 ACS patients were pre-screened at that time; in the first step, due to current expert recommendations [9], we excluded patients with STEMI. Out of all remaining ACS cases (*n* = 3310) following assessment using the inclusion and exclusion criteria, we selected the study population. Figure 1 presents a flow chart of the study design

Patients with diabetes mellitus (*n* = 72) were compared with a non-diabetic group (*n* = 121). Clinical follow-up was obtained on the 30th day and after 1 year following the office visits or telephone interviews. All of the data obtained using a standardized questionnaire were collected by trained medical staff and entered retrospectively into an electronic database.

### 2.2. Device and Procedures

The Magmaris is a second-generation metallic (magnesium) sirolimus-eluting bioresorbable scaffold containing an active bioabsorbable coating—BIOlute Poly-L-Lactide (PLLA)—with controlled drug release up to 90 days and with an average time of full scaffold absorption amounting to approximately one year. The Magmaris used in this study was available in diameters of 3.0 and 3.5 mm and lengths of 15, 20, and 25 mm. The decision to perform PCI was based on current guidelines for ACS management. The selection of lesions qualified for treatment with Magmaris was carried out in accordance with the current recommendations and with the consensus of experts [9]. All implantations were performed with the 4P strategy, which includes patient selection (de novo lesions with a vessel diameter and lesion length matching Magmaris sizes); proper sizing (reference vessel diameter in a range from 2.7 mm up to 3.7 mm); pre-dilatation (mandatory with a non-compliant (NC) balloon-sized 1:1 balloon-to-artery ratio, without significant residual stenosis); and post-dilatation (mandatory, high-pressure (not less than 16 atm) with NC balloon-sized 1:1 balloon/scaffold ratio or up to 0.5 mm longer). The decision to use intravascular imaging for guidance was left to the operators. Standard pharmacotherapy was followed according to the current ESC/ESH guidelines for non-ST segment elevation myocardial infarct (NSTEMI) [10,11], and dual antiplatelet therapy lasted 12 months.

### 2.3. Endpoints and Definitions

The primary outcome was a safety composite of cardiovascular death, myocardial infarction, and definite or probable in-stent thrombosis at 30-days and 1-year follow-up. The principal secondary outcome was an effectiveness outcome of target-lesion failure (TLF) defined as cardiac death, target vessel myocardial infarction (TV-MI), or target lesion revascularization (TLR). Other secondary outcomes included scaffold restenosis, death from any reason, cerebrovascular episodes, revascularization procedures, as well as myocardial infarction, defined according to the Fourth Universal Definition of Myocardial Infarction [12].

### 2.4. Statistical Analysis

The analyses were conducted using the R language [13]. Continuous variables were characterized with their mean and standard deviation, while frequencies were used for categorical variables. The patients were compared between groups with the nonparametric two-sample Mann–Whitney’s test for continuous variables and Fisher’s Exact Test for categorical variables. Bonferroni correction was applied to adjust for multiple comparisons. *p*-values ≤ 0.05 were accepted as a threshold for statistical significance.

## 3. Results

Following the inclusion and exclusion criteria, finally, a total of 193 patients with acute coronary syndrome were enrolled in this study and their baseline clinical characteristic is shown in Table 1. Between October 2016 and March 2020, *n* = 72 DM patients with ACS underwent PCI with 74 BRS implantation, of which 58 (80.5%) patients were treated with oral medication and 14 (19.5%) patients were treated with insulin. The majority of patients were male (80.5%), with NSTEMI as an initial clinical manifestation (80.5%). The anatomical complexity of lesions in both groups was relatively low-type A/B1 lesions dominated in the diabetic population (77.8%) and the control group (80.9%).

The success rate for device implantation (204 Magmaris BRS) was 100%. There were 121 patients in the control non-diabetic group. Additionally, this group consisted mainly of males (76%) with a clinical diagnosis of NSTEMI (86.7%). The diabetic group had a significantly higher prevalence of hypertension and a past history of PCI. In contrast, a non-diabetic group had significantly higher serum lipid levels as well as left ventricular ejection fraction. All procedural-related data are presented in Table 2; postprocedural TIMI grade 3 was observed after all procedures.

In the diabetic group between 1–12 months after the index PCI procedure, two fatal stroke cases were recognized compared to one non-fatal TIA episode in the control group. No scaffold thrombosis occurred during the observation period; however, two cases of in-stent restenosis with one ACS-NSTEMI case were recognized in the DM group. Seven diabetic patients were scheduled for stage procedures of a non-culprit vessel after the index PCI. During those procedures, one case of asymptomatic scaffold restenosis was diagnosed. For comparison in the non-diabetic group, six patients underwent scheduled PCI. The one-year follow-ups did not show any significant differences regarding primary outcome (cardiac death, myocardial infarction, and stent thrombosis). In the diabetic group, we found a higher rate of principal secondary outcome (just beyond statistical significance *p* = 0.051). It is connected with the fact that diabetic patients were more prone to experience target vessel revascularization (*p* = 0.051) and target vessel myocardial infarct (*p* = 0.138). Table 3 summarizes the 30-day and 1-year follow-ups.

## 4. Discussion

Diabetes mellitus (DM) is one of the major risk factors increasing mortality in the course of ACS. Diabetic patients more often presented with NSTEMI. Moreover, due to comorbidities, they less frequently undergo percutaneous coronary angioplasty [14]. Despite advancements in pharmacologic agents and devices, PCI in the diabetic population is still associated with a higher rate of adverse events [15]. Multivessel CAD remains a strong indication for surgical treatment and even intensive glucose control after PCI does not improve the clinical outcome [16]. Increased rates of restenosis and stent thrombosis [17] are related to a chronic inflammatory response that accelerates neointimal hyperplasia and promotes platelet activation and adhesion. To minimize these phenomena, the bioresorbable scaffold had been developed. Theoretically, BRS provides a short-term vessel patency equivalent to the DES and offers complete reabsorption of the scaffold within specified time frames. Thus far, there are no data capable of evaluating the outcome of Magmaris in diabetes patients.

The data related to BRS implantation in diabetic patients are mainly based on the studies dedicated to the Absorb scaffold. In view of the preliminary encouraging results [18], Absorb (Abbott Vascular, Santa Clara, CA, USA) scaffolds have become the most frequently implanted BRS. Additionally, the short-term data regarding the use of Absorb BVS in anatomically low-risk patients with DM was favorable [19] and revealed acceptable safety and efficacy outcomes at 1-year, which is consistent with our results obtained after Magmaris implantation. Analogous to our data, the previously mentioned Absorb registry included mainly single, large-diameter BVS implanted with subsequent aggressive post-dilation. In our registry, over 80% of all implanted BRS was post-dilatated over the nominal scaffold size. It seems to be crucial in obtaining satisfactory angioplasty results.

The AIDA-trial sub-study focusing on the diabetes population long-term follow-up [20] also did not reveal a significant difference in the clinical events between DES and Absorb yet revealed a higher rate of device-related thrombosis in the Absorb group. This study confirmed the negative impact of diabetes on the prognosis of patients after PCI regardless of the type of scaffold used.

However, due to the unfavorable long-term outcomes in the Absorb II [5] and Absorb III studies [21], the widespread use of this BVS was suspended. The main recognized reasons for these compromised safety outcomes included insufficient lesion preparation; under-sizing; increased loss of vessel diameter along with the distal device implantation; asymmetric or heterogeneous degradation; neo-atherosclerosis, leading finally to an increased rate of in-stent restenosis; and acute or late stent thrombosis [22,23] All of these phenomena are particularly pronounced in the low-diameter stents (less than 3 mm) [9]. Therefore, the “4P technique“ (patient selection, proper sizing, pre-dilatation, and post-dilatation strategy) was proposed for appropriate stent optimization and was an essential part of the BRS implantation procedure in this study. This optimal implantation technique allowed us to partially reset the negative impact of diabetes on patients treated with BRS-ABSORB implantation [24,25]. However, the rate of peri-procedural complications remained higher than that in DES. In our study, we did not observe a similar correlation. Particularly important seems to be a safety concern related to the thrombotic issue. Pooled data from the Absorb studies [26] showed an increased amount of device thrombosis in relation to “classical” drug-eluting stents. Diabetes, especially treated with insulin, is a well-established risk factor of Absorb device thrombosis [27].

Our registry does not confirm the observations that the first generation of BRS increased the rate of acute and late thrombosis even in the diabetic population. These data appear to be consistent with the outcomes of a preclinical study [28]. Noteworthily, in our registry consisting of ACS cases (extended thrombotic process), none of the stent thrombosis in a 1-year period occurred. However, there is a noticeable trend of an increased rate of in-stent restenosis in the diabetic group leading to an increased rate of target lesion failure. However, both effects are without statistical significance. Towards, it resulted in an increased rate of principal secondary outcomes related to a statistically insignificant higher number of target vessel revascularization and target vessel myocardial infarct. Moreover, subsequent studies with a higher number of patients and a longer period of observation are needed in order to define this trend as clinically relevant. However, taking into account the data collected for DES studies [29,30], it seems promising.

Our favorable results in both the diabetic and non-diabetic groups might be also connected to the itemized lesion selection. We avoided lesions with challenging tortuosity or angulation, heavy calcification, or thrombus presence. We exercised cautious in lesion selection regarding the Magmaris manufacturer’s recommendations as well as unfavorable experience with the first-generation BRS. The COMPARE-ABSORB trial [31] randomly recruited high-risk in-stent restenosis (BVS or DES) patients and was discontinued prematurely due to a high rate of scaffold thrombosis and TVMI in the BRS arm.

### Limitations

This was a single-center, non-randomized study with retrospective data collection in the relatively short observation period (1-year follow-up). The study population was not very large, and the rate of intravascular guidance PCI in this study was comparatively low.

## 5. Conclusions

To the best of our knowledge, this is the first human study designed to demonstrate the efficacy and safety of Magmaris, a novel magnesium bioresorbable scaffold, in a diabetic population in ACS settings. Our early outcome seems to be favorable and suggests that this novel BRS is safe and effective in low-risk patients with ACS and concomitant DM. Nevertheless, there is a strong need for large multicenter, randomized, prospective studies with a longer observation period in order to perform a full assessment of this novel device in diabetic patients with ACS. 

## Figures and Tables

**Table 1:** Baseline clinical characteristic of both groups.

	Diabetes Patients N = 72	Non-Diabetes Patients N = 121	*p*-Value
Age	65.3 ± 7.9	63.2 ± 9.5	*p* = 0.127
Gander—male (ratio)	58 (80.5%)	92 (76.0%)	*p* = 0.592
Unstable angina	14 (19.5%)	16 (13.2%)	*p* = 0.305
NSTEMI	58 (80.5%)	105 (86.7%)	*p* = 0.305
Oral anti-diabetic Treatment	58 (80.5%)	NA	-
Insulin	14 (19.5%)	NA	-
Hypertension	69 (95.8%)	102 (84.2%)	***p* = 0.018**
Hyperlipidemia	58 (80.5%)	94 (77.0%)	*p* = 0.718
Atrial Fibrillation	2 (2.7%)	7 (5.7%)	*p* = 0.488
Previously PCI	36 (50%)	42 (34.7%)	***p* = 0.048**
Primary Diagnosis MI	28 (38.8%)	31 (25.6%)	*p* = 0.075
Current smoker	22 (30.5%)	35 (28.9%)	*p* = 0.871
LV-EF	57.7% ± 10.7	59.4% ± 16.0	***p* = 0.050**
Total Cholesterol (mmol/L)	4.3 ± 1.3	4.8 ± 1.3	***p* = 0.008**
LDL (mmol/L)	2.1 ± 0.9	2.8 ± 1.2	***p* < 0.001**
Triglycerides (mmol/L)	1.9 ± 1.1	1.8 ± 2.1	*p* = 0.213
Creatine (µmol/L)	82.3 ± 21.5	85.1 ± 22.5	*p* = 0.431
Days of hospitalization	2.9 ± 2.0	2.7 ± 1.6	*p* = 0.866

Abbreviations: NSTEMI—non-ST elevation myocardial infraction; PCI—percutaneous coronary intervention; MI—myocardial infraction; LV-EF-LV—left ventricular ejection fraction. Bold data- mark statistically significant (*p* < 0.05) differences between two groups. Bold data mark statistically significant differences.

**Table 2:** Procedural characteristic of both groups.

	Diabetes Patients N = 72	Non-Diabetes Patients N = 121	*p*-Value
Treated vessel:			
LAD	31 (43%)	49 (40.5%)	*p* > 0.999
LCx	18 (25%)	31 (25.6%)	*p* > 0.999
RCA	22 (30.6%)	39 (32.2%)	*p* > 0.999
IM	1 (1.4%)	2 (1.7%)	*p* > 0.999
Predilatation balloon:			
Mean diameter (mm)	3.20 ± 0.24	3.24 ± 0.27	*p* = 0.273
Mean pressure (atm)	17.75 ± 0.75	17.57 ± 0.91	*p* = 0.209
Average scaffold number	1.03 ± 0.17	1.07 ± 0.26	*p* = 0.179
Scaffold diameter:			
3.0 (mm)	35 (47.2%)	53 (40.7%)	*p* = 0.552
3.5 (mm)	39 (52.7%)	77 (59.3%)	*p* = 0.225
Average scaffold length (mm)	21.11 ± 3.27	20.62 ± 3.26	*p* = 0.308
Post-dilatation balloon:			
-Mean diameter (mm)	3.51 ± 0.31	3.55 ± 0.29	*p* = 0.495
-Mean pressure (atm)	17.69 ± 0.80	17.72 ± 0.83	*p* = 0.924
-0.0 mm greater than scaffold	12 (16.6%)	19 (15.7%)	*p* = 0.843
-0.25 mm greater than scaffold	47 (65.2%)	83 (68.6%)	*p* = 0.638
-0.5 mm greater than scaffold	13 (18.2%)	19 (15.7%)	*p* = 0.692
Syntax Score	7.7 ± 4.2	7.5 ± 4.5	-
AHA/ACC classification type:			
A/B1	56 (77.8%)	98 (80.9%)	*p* = 0.871
B2/C	16 (22.2%)	23 (19.1%)	*p* = 0.866
Contrast Volume (mL)	153.22 ± 76.76	150.21 ± 57.64	*p* = 0.337
Dose of radiation (mGy)	1120.18 ± 843.89	1014.70 ± 591.75	*p* = 0.934
OCT-guided PCI	13 (18%)	28 (23.1%)	*p* = 0.469
Number of edge dissection:	3 (4.1%)	4 (3.3%)	*p* = 0.713
-treated with Magmaris	0 (0%)	3 (2.4%)	*p* = 0.295
-treated with DES	3 (4.1%)	1 (0.8%)	*p* = 0.147
Side branch occlusion	0 (0%)	2 (1.6%)	*p* = 0.530
Antiplatelet Drug:			
ASA	72 (100%)	121 (100%)	-
Clopidogrel	26 (36.1%)	50 (41.3%)	*p* = 0.543
Ticagrelor	46 (63.9%)	71 (58.7%)	*p* = 0.543

Abbreviations: OCT—optical coherence tomography; PCI—percutaneous coronary intervention; ASA—acetylsalicylic acid; AHA—American Heart Association; ACC—American College of Cardiology.

**Table 3:** Clinical outcomes.

	Diabetes Patients N = 72	Non-Diabetes Patients N = 121	*p*-Value
30-Day FU Primary outcome: cardiac death, myocardial infarction, stent thrombosis	0 (0%)	0 (0%)	-
30-Day FU Principal secondary outcome: Target lesion failure (cardiac death, target vessel myocardial infract, target lesion-revascularization)	0 (0%)	0 (0%)	-
30-Day FU Death:			
-Any death	0 (0%)	0 (0%)	-
-Cardiac death	0 (0%)	0 (0%)	-
30-Day FU Myocardial infraction:			
-Any MI	0 (0%)	0 (0%)	-
-Target vessel myocardial infract	0 (0%)	0 (0%)	-
30-Day FU Scaffold thrombosis	0 (0%)	0 (0%)	-
Scaffold restenosis	0 (0%)	0 (0%)	-
30-Day FU Stroke	0 (0%)	0 (0%)	-
TIA	0 (0%)	0 (0%)	-
30-Day FU Revascularization:			
-Target lesion revascularization	0 (0%)	0 (0%)	-
-Target vessel revascularization	0 (0%)	0 (0%)	-
-Any revascularization	0 (0%)	0 (0%)	-
1-Year FU Primary outcome: cardiac death, myocardial infarction, stent thrombosis	2 (2.7%)	1 (0.8%)	*p* = 0.557
1-Year FU Principal secondary outcome: Target lesion failure (cardiac death, target vessel myocardial infract, target lesion-revascularization)	3 (4.1%)	0 (0%)	*p* = 0.051
1-Year FU Death			
-Any death	2 (2.7%)	0(0%)	*p* = 0.138
-Cardiac death	0 (0%)	0(0%)	*-*
1-Year FU Myocardial infraction:			
-Any MI	2 (2.7%)	1 (0.8%)	*p* = 0.557
-Target vessel myocardial infract	2 (1.3%)	0 (0%)	*p* = 0.138
1-Year FU Scaffold thrombosis	0 (0%)	0 (0%)	-
Scaffold restenosis	2 (2.7%)	0 (0%)	*p* = 0.138
1-Year FU Stroke	2 (2.7%)	0 (0%)	*p* = 0.138
TIA	0 (0%)	1 (0.8%)	*p* > 0.999
1-Year FU Revascularization:			
-Target lesion revascularization	2 (2.7%)	0 (0%)	*p* = 0.138
-Target vessel revascularization	3 (2.7%)	0 (0%)	*p* = 0.051
-Any revascularization	10 (13.8%)	8 (6.6%)	*p* = 0.124

Abbreviations: TIA—transient ischemic attack; PCI—percutaneous coronary intervention; ASA—acetylsalicylic acid; MI—myocardial infraction.

## Data Availability

Data not included in manuscript available on request from corresponding author due to local law and privacy restrictions.

## References

[1] Shaw J.E., Sicree R.A., Zimmet P.Z. (2010). Global estimates of the prevalence of diabetes for 2010 and 2030. Diabetes Res. Clin. Pract..

[2] Wang H., Gao Z., Song Y., Tang X., Xu J., Jiang P., Jiang L., Chen J., Gao L., Song L. (2018). Impact of diabetes mellitus on percutaneous coronary intervention in Chinese patients: A large single-center data. Angiology.

[3] Nakazawa G., Finn A.V., Virmani R. (2007). Vascular pathology of drug-eluting stents. Herz Kardiovaskuläre Erkrankungen..

[4] Joner M., Finn A.V., Farb A., Mont E.K., Kolodgie F.D., Ladich E., Kutys R., Skorija K., Gold H.K., Virmani R. (2006). Pathology of drug-eluting stents in humans: Delayed healing and late thrombotic risk. J. Am. Coll. Cardiol..

[5] Serruys P.W., Chevalier B., Sotomi Y., Cequier A., Carrié D., Piek J.J., Van Boven A.J., Dominici M., Dudek D., McClean D. (2016). Comparison of an everolimus-eluting bioresorbable scaffold with an everolimus-eluting metallic stent for the treatment of coronary artery stenosis (ABSORB II): A 3 year, randomised, controlled, single-blind, multicentre clinical trial. Lancet.

[6] Wlodarczak A., Garcia L.A., Karjalainen P.P., Komócsi A., Pisano F., Richter S., Lanocha M., Rumoroso J.R., Leung K.F. (2019). Magnesium 2000 postmarket evaluation: Guideline adherence and intraprocedural performance of a sirolimus-eluting resorbable magnesium scaffold. Cardiovasc. Revasc. Med..

[7] Wlodarczak A., Lanocha M., Jastrzebski A., Pecherzewski M., Szudrowicz M., Jastrzebski W., Nawrot J., Lesiak M. (2019). Early outcome of magnesium bioresorbable scaffold implantation in acute coronary syndrome—The initial report from the Magmaris-ACS registry. Catheter. Cardiovasc. Interv..

[8] Capranzano P., Capodanno D., Brugaletta S., Latib A., Mehilli J., Nef H., Gori T., Lesiak M., Geraci S., Pyxaras S. (2018). Clinical outcomes of patients with diabetes mellitus treated with Absorb bioresorbable vascular scaffolds: A subanalysis of the E uropean M ulticentre GHOST-EU R egistry. Catheter. Cardiovasc. Interv..

[9] Fajadet J., Haude M., Joner M., Koolen J., Lee M., Tölg R., Waksman R. (2016). Magmaris preliminary recommendation upon commercial launch: A consensus from the expert panel on 14 April 2016. EuroIntervention.

[10] Collet J.P., Thiele H., Barbato E., Barthélémy O., Bauersachs J., Bhatt D.L., Dendale P., Dorobantu M., Edvardsen T., Folliguet T. (2021). 2020 ESC Guidelines for the management of acute coronary syndromes in patients presenting without persistent ST-segment elevation: The Task Force for the management of acute coronary syndromes in patients presenting without persistent ST-segment elevation of the European Society of Cardiology (ESC). Eur. Heart J..

[11] Valgimigli M., Bueno H., Byrne R.A., Collet J.P., Costa F., Jeppsson A., Jüni P., Kastrati A., Kolh P., Mauri L. (2018). 2017 ESC focused update on dual antiplatelet therapy in coronary artery disease developed in collaboration with EACTS. Eur. J. Cardiothorac. Surg..

[12] Thygesen K., Alpert J.S., Jaffe A.S., Chaitman B.R., Bax J.J., Morrow D.A., White H.D., Executive Group on behalf of the Joint European Society of Cardiology (ESC), American College of Cardiology (ACC), American Heart Association (AHA) (2018). Fourth universal definition of myocardial infarction (2018). J. Am. Coll. Cardiol..

[13] R Core Team (2020). R: A language and Environment for Statistical Computing: R Foundation for Statistical Computing.

[14] Alabas O.A., Hall M., Dondo T.B., Rutherford M.J., Timmis A.D., Batin P.D., Deanfield J.E., Hemingway H., Gale C.P. (2017). Long-term excess mortality associated with diabetes following acute myocardial infarction: A population-based cohort study. J. Epidemiol. Community Health.

[15] Xin X., Wang X., Dong X., Fan Y., Shao W., Lu X., Xiao P. (2019). Efficacy and safety of drug-eluting stenting compared with bypass grafting in diabetic patients with multivessel and/or left main coronary artery disease. Sci. Rep..

[16] Zhai C., Cong H., Hou K., Hu Y., Zhang J., Zhang Y. (2019). Clinical outcome comparison of percutaneous coronary intervention and bypass surgery in diabetic patients with coronary artery disease: A meta-analysis of randomized controlled trials and observational studies. Diabetol. Metab. Syndr..

[17] Zhuo X., Zhang C., Feng J., Ouyang S., Niu P., Dai Z. (2019). In-hospital, short-term and long-term adverse clinical outcomes observed in patients with type 2 diabetes mellitus vs non-diabetes mellitus following percutaneous coronary intervention: A meta-analysis including 139,774 patients. Medicine.

[18] Ellis S.G., Kereiakes D.J., Metzger D.C., Caputo R.P., Rizik D.G., Teirstein P.S., Litt M.R., Kini A., Kabour A., Marx S.O. (2015). Everolimus-eluting bioresorbable scaffolds for coronary artery disease. N. Engl. J. Med..

[19] Hommels T.M., Hermanides R.S., Rasoul S., Berta B., IJsselmuiden A.J., Jessurun G.A., Benit E., Pereira B., De Luca G., Kedhi E. (2019). The 1-year safety and efficacy outcomes of Absorb bioresorbable vascular scaffolds for coronary artery disease treatment in diabetes mellitus patients: The ABSORB DM Benelux study. Neth. Heart J..

[20] Tijssen R.Y., van der Schaaf R.J., Kraak R.P., Vink M.A., Hofma S.H., Arkenbout E.K., Weevers A.P., Kerkmeijer L.S., Onuma Y., Serruys P.W. (2020). Clinical outcomes at 2 years of the Absorb bioresorbable vascular scaffold versus the Xience drug-eluting metallic stent in patients presenting with acute coronary syndrome versus stable coronary disease—AIDA trial substudy. Catheter. Cardiovasc. Interv..

[21] Kereiakes D.J., Ellis S.G., Metzger C., Caputo R.P., Rizik D.G., Teirstein P.S., Litt M.R., Kini A., Kabour A., Marx S.O. (2017). 3-year clinical outcomes with everolimus-eluting bioresorbable coronary scaffolds: The ABSORB III trial. J. Am. Coll. Cardiol..

[22] Puricel S., Cuculi F., Weissner M., Schmermund A., Jamshidi P., Nyffenegger T., Binder H., Eggebrecht H., Münzel T., Cook S. (2016). Bioresorbable coronary scaffold thrombosis: Multicenter comprehensive analysis of clinical presentation, mechanisms, and predictors. J. Am. Coll. Cardiol..

[23] Stone G.W., Abizaid A., Onuma Y., Seth A., Gao R., Ormiston J., Kimura T., Chevalier B., Ben-Yehuda O., Dressler O. (2017). Effect of technique on outcomes following bioresorbable vascular scaffold implantation: Analysis from the ABSORB trials. J. Am. Coll. Cardiol..

[24] Hommels T.M., Hermanides R.S., Rasoul S., Berta B., IJsselmuiden A.J., Jessurun G.A., Benit E., Pereira B., De Luca G., Kedhi E. (2019). Everolimus-eluting bioresorbable scaffolds for treatment of coronary artery disease in patients with diabetes mellitus: The midterm follow-up of the prospective ABSORB DM Benelux study. Cardiovasc. Diabetol..

[25] Anadol R., Schnitzler K., Lorenz L., Weissner M., Ullrich H., Polimeni A., Münzel T., Gori T. (2018). Three-years outcomes of diabetic patients treated with coronary bioresorbable scaffolds. BMC Cardiovasc. Disord..

[26] Stone G.W., Kimura T., Gao R., Kereiakes D.J., Ellis S.G., Onuma Y., Chevalier B., Simonton C., Dressler O., Crowley A. (2019). Time-varying outcomes with the absorb bioresorbable vascular scaffold during 5-year follow-up: A systematic meta-analysis and individual patient data pooled study. JAMA Cardiol..

[27] Kereiakes D.J., Ellis S.G., Kimura T., Abizaid A., Zhao W., Veldhof S., Vu M.T., Zhang Z., Onuma Y., Chevalier B. (2017). Efficacy and safety of the absorb everolimus-eluting bioresorbable scaffold for treatment of patients with diabetes mellitus: Results of the absorb diabetic substudy. JACC Cardiovasc. Interv..

[28] Waksman R., Lipinski M.J., Acampado E., Cheng Q., Adams L., Torii S., Gai J., Torguson R., Hellinga D.M., Westman P.C. (2017). Comparison of acute thrombogenicity for metallic and polymeric bioabsorbable scaffolds: Magmaris versus absorb in a porcine arteriovenous shunt model. Circ. Cardiovasc. Interv..

[29] Kim M.S., Dean L.S. (2011). In-stent restenosis. Cardiovasc. Ther..

[30] Januszek R.A., Dziewierz A., Siudak Z., Rakowski T., Legutko J., Rzeszutko Ł., Kleczyński P., Dudek D., Bartuś S. (2020). Diabetes and periprocedural outcomes in patients treated with rotablation during percutaneous coronary interventions. Cardiol. J..

[31] Smits P.C., Chang C.C., Chevalier B., West N.E., Anadol R., Barbato E., Tarantini G., Kocka V., Achenbach S., Dudek D. (2020). Bioresorbable Vascular Scaffold Versus Metallic Drug-Eluting Stent in Patients at High Risk of Restenosis: The COMPARE-ABSORB Randomized Clinical Trial. Randomized Control. Trial.

